# Supplementary figures and images for: Peroxisomal dysfunctions cause lysosomal storage and axonal Kv1 channel redistribution in peripheral neuropathy
Source: eLife. 2017 May 4;6:e23332. doi: 10.7554/eLife.23332 (PMC5417850; doi:10.7554/eLife.23332)

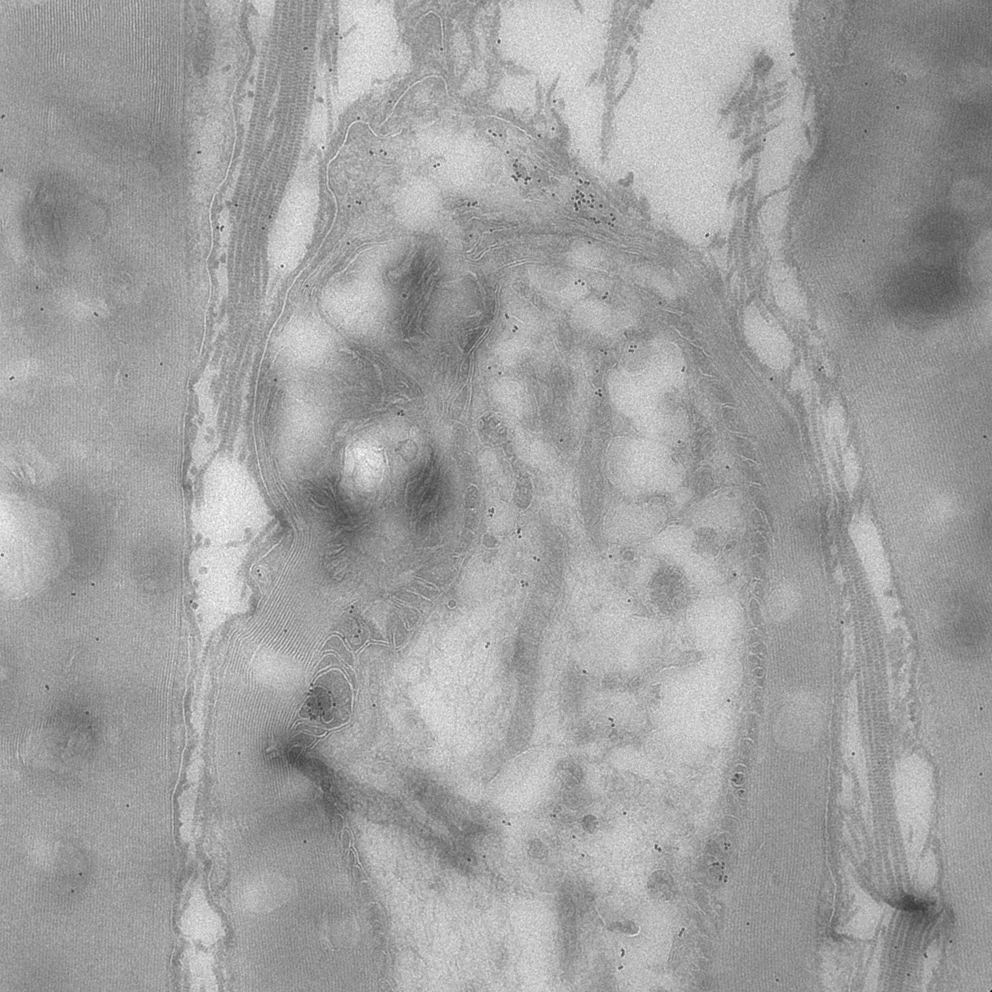

Supplement: Figure 3—source data 1. — DOI: http://dx.doi.org/10.7554/eLife.23332.012 [file elife-23332-fig3-data1.zip › Fig 3 comp/16828.tif]

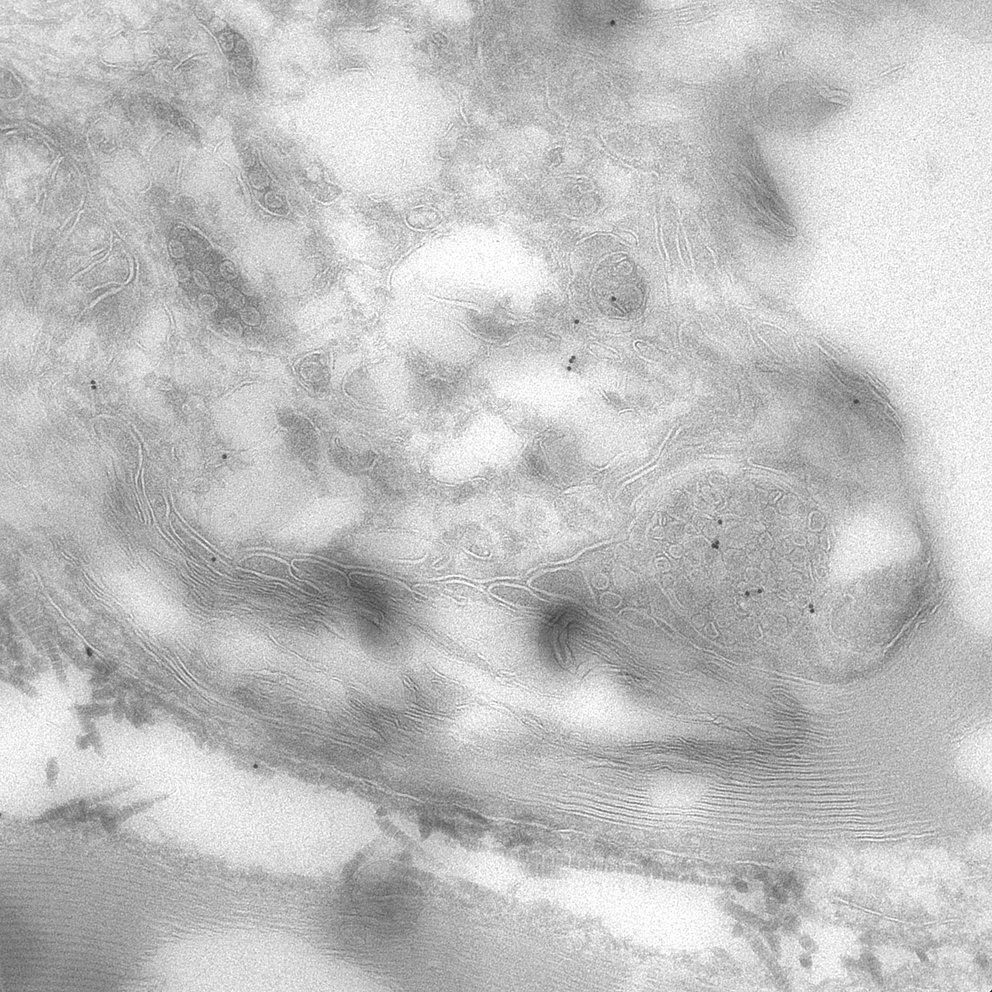

Supplement: Figure 3—source data 1. — DOI: http://dx.doi.org/10.7554/eLife.23332.012 [file elife-23332-fig3-data1.zip › Fig 3 comp/16837.tif]
