# Supplementary material for: Peroxisomal dysfunctions cause lysosomal storage and axonal Kv1 channel redistribution in peripheral neuropathy
Source: eLife. 2017 May 4;6:e23332. doi: 10.7554/eLife.23332 (PMC5417850; doi:10.7554/eLife.23332)
Supplement: Figure 3—source data 1. — DOI: http://dx.doi.org/10.7554/eLife.23332.012 [file elife-23332-fig3-data1.zip › Fig 3 comp/Figure 3_Source Data 1.doc]

**Figure 3–Source Data 1. Lysosomal accumulation within paranodal loops.**

Immuno electron micrographs of sciatic nerves obtained from four individual PEX5 mutant animals at age >9 months using anti-LAMP1 antibody showing the paranodal accumulation of lysosomes.
